# Karyotype diversity and 2C DNA content in species of the *Caesalpinia* group

**DOI:** 10.1186/s12863-018-0610-2

**Published:** 2018-04-11

**Authors:** Polliana Silva Rodrigues, Margarete Magalhães Souza, Cláusio Antônio Ferreira Melo, Telma Nair Santana Pereira, Ronan Xavier Corrêa

**Affiliations:** 1 Departamento de Ciências Biológicas, Centro de Biotecnologia e Genética, Universidade Estadual de Santa Cruz, Rodovia Jorge Amado, km 16, CEP, Ilhéus, BA 45662-900 Brazil; 2 Centro de Ciências e Tecnologias Agropecuárias, Laboratório de Melhoramento Genético Vegetal, Universidade Estadual do Norte Fluminense, Campos dos Goytacazes, Brazil

**Keywords:** Caesalpinioideae, Flow cytometry, CMA_3_^+^/DAPI^−^, Fluorescent in situ hybridization, Pau-Brasil

## Abstract

**Background:**

The Leguminosae family is the third-largest family of angiosperms, and Caesalpinioideae is its second-largest subfamily. A great number of species (approximately 205) are found in the *Caesalpinia* group within this subfamily; together with these species’ phenotypic plasticity and the similarities in their morphological descriptors, make this a complex group for taxonomic and phylogenetic studies. The objective of the present work was to evaluate the karyotypic diversity and the 2C DNA content variation in 10 species of the *Caesalpinia* group, representing six genera: *Paubrasilia*, *Caesalpinia, Cenostigma*, *Poincianella*, *Erythrostemon* and *Libidibia*. The GC-rich heterochromatin and 45S rDNA sites (which are used as chromosome markers) were located to evaluate the karyotype diversity in the clade. The variation in the 2C DNA content was determined through flow cytometry.

**Results:**

The fluorochrome banding indicated that the chromomycin A_3_^+^/4′,6-diamidino-2-phenylindole^−^ blocks were exclusively in the terminal regions of the chromosomes, coinciding with 45S rDNA sites in all analyzed species. Physical mapping of the species (through fluorescence in situ hybridization) revealed variation in the size of the hybridization signals and in the number and distribution of the 45S rDNA sites. All hybridization sites were in the terminal regions of the chromosomes. In addition, all species had a hybridization site in the fourth chromosome pair. The 2C DNA content ranged from 1.54 pg in *Erythrostemon calycina* to 2.82 pg in the *Paubrasilia echinata* large-leaf variant. The *Pa. echinata* small-leaf variant was isolated from the other leaf variants through Scoot-Knott clustering.

**Conclusions:**

The chromosome diversity and the variation in the 2C DNA content reinforce that the actual taxonomy and clustering of the analyzed taxa requires more genera that were previously proposed. This fact indicates that taxonomy, phylogeny and cytoevolutionary inference related to the complex *Caesalpinia* group have to be done through integrative evaluation.

## Background

Leguminosae is the third-largest family among angiosperms [1], and Caesalpinioideae, its second-largest subfamily, is represented by about 170 genera, many of them with complex and confused taxonomies. This subfamily’s phenotypic plasticity is a challenge for taxonomies that are based on morphology [2]. This group commonly occurs in Brazil, which is home to about 790 described species [3, 4]. The *Caesalpinia* group within the Caesalpinioideae subfamily is a pantropical clade that presents about 205 pantropical species [5], including important Brazilian species that are threatened with extinction [6].

Taxonomic and phylogenetic changes have been done for some species and genera from Leguminosae, including the clustering of taxa in a new genetic system for the *Caesalpinia* group [5]. The major problem in the *Caesalpinia* clade taxonomy and its phylogenetic classification relates to morphological similarities, as there is little variation for some descriptors [7]. Solving this problem requires a broad mode of analysis for the taxa characterization [8, 9], which has been helpful for the systematic distribution and taxonomy of the *Caesalpinia* group [10, 11].

The banding obtained from the application of chromomycin A_3_ (CMA_3_) and 4′,6-diamidino-2-phenylindole (DAPI) fluorochromes, and from the localization of 45S rDNA sites using the fluorescence in situ hybridization (FISH) technique, has been used to identify specific sites; the positions and sizes of such tags may be useful as cytological markers. These data allow us to define the location and quantity of the chromosome markers that are commonly observed in a group of species, as well as the specific chromosome pattern of the markers for each species [12].

Flow cytometry has been used in biosystematics analyses, mainly to provide results regarding nuclear DNA content and, consequently, the level of ploidy. This allows for better species detection and delimitation, which is helpful in the study of a particular genus’s phylogenetic relationships and evolutionary characteristics [13].

Previous studies in which fluorochrome staining was applied to the Caesalpinioideae subfamily revealed inter- and intraspecific differences. The heterochromatic blocks observed (CMA_3_^+^/DAPI^−^) were distributed in regions proximal to the nuclear organizer regions, but the presence of CMA_3_^+^/DAPI^−^ blocks was also observed in previous studies [14]. The DNA content indicated the existence of intra- and interspecific variability in some genera within Fabaceae [15–17]. However, these analyses included only one DNA-content analysis for a species of the genus *Caesalpinia* (*Caesalpinia crista*) [17].

This study aimed to evaluate the karyotype diversity in 10 species (representing six genera) of the pantropical *Caesalpinia* clade, using the location of GC-rich heterochromatin and the number and position of 45S rDNA sites. In addition, 2C DNA content was quantified using flow cytometry.

## Methods

### Botanical material and pretreatment

Seeds of 10 species of the *Caesalpinia* group were collected from several locations in the state of Bahia in Brazil (Table 1). The seeds were randomly collected, with the name of obtaining as many species from the state as possible. After field collection, the seeds were treated with Captan (Fersol®) fungicide and germinated on moistened filter paper in a humid chamber at room temperature. Root tips of approximately 3 mm in length were collected shortly after germination and pretreated with an anti-mitotic solution of 0.002 M 8-hydroxyquinoline for 6 h; the root tips were then washed twice in distilled water, dried on filter paper fixed in Carnoy I (3:1 glacial acetic acid to absolute ethanol, *v*/v) [18] for 2 h at room temperature, and maintained at − 20 °C until the time of use. After radicles were collected, the seedlings were planted in 2 kg bags with organic soil and monitored in a greenhouse, resulting in matrix plants for the cytogenetic characterization.

**Table 1:** Estimates of nuclear genome size (2C DNA content) for *Caesalpinia* group

Species	Collect site	2C (pg)^a^	CV %
*Paubrasilia echinata* (Lam.) E. Gagnon, H.C. Lima & G.P. Lewis SV (pau-brasil or brazilwood)	Ilhéus/BA 14° 39′ 09” S. 39° 10′ 10” Wo.	2.76^B^	5.25
*Pa. echinata* MV^b^	Una/BA 15° 17′ 36” S. 39° 04′ 31” Wo.	2.81^A^	4.67
*Pa. echinata* LV^b^	Una/BA 15° 17′ 36” S. 39° 04′ 31” Wo.	2.82^A^	4.52
*Caesalpinia pulcherrima* (L.) Sw.	Ilhéus/BA 14° 39′ 09” S. 39° 10′ 10” Wo.	1.63^E^	5.80
*Cenostigma macrophyllum* Tul.	Ibotirama/BA 12° 0.9′ 19.5” S. 43° 10′ 03.9” Wo.	1.83^D^	6.65
*Poincianella pyramidalis* (Tul.) L. P. Queiroz	Bom Jesus da Lapa/BA 13° 19′ 0.9” S. 43° 20′ 14.8” Wo.	1.92^C^	8.03
*Po. laxiflora* (Tul.) L.P. Queiroz	Bom Jesus da Lapa/BA 13° 19′ 0.9” S. 43° 20′ 14.8” Wo.	1.90^C^	6.65
*Po. microphylla* (Mart. ex. G. Don) L.P. Queiroz	Xique-Xique/BA 10° 49′ 19” S. 42° 43′ 51” Wo.	1.88^C^	7.35
*Po. pluviosa* (DC.) L. P. Queiroz	Ilhéus/BA 14° 39′ 09” S. 39° 10′ 10” Wo.	1.87^C^	6.28
*Po. bracteosa* (Tul.) L.P. Queiroz	Oliveira dos Brejinhos/BA 12° 19′ 01” S. 42° 53′ 45” Wo.	1.92^C^	5.95
*Erythrostemon calycina* (Benth.) L.P. Queiroz	Livramento de Nossa Senhora/BA 13° 39′ 07.6” S. 41° 50′ 45.9” Wo.	1.54^F^	6.75
*Libidibia ferrea* (Mart. Ex Tul.) L.P. Queiroz	Ilhéus/BA 14° 39′ 09” S. 39° 10′ 10” Wo.	1.60^E^	9.53

(CV%) Coefficient of variation

^a^The averages for the 2C DNA followed by the same letter did not differ statistically from one another by the Scott-Knott group at 0.05% with DMS: 0.0647

^b^During the duration of this study seeds of the medium and large variants have not been found and cytometry analyses were performed on seedlings obtained by donation

### Preparation of slides and banding with CMA_3_ and DAPI fluorochromes

For the localization of base regions specific to GC and AT, the fluorochromes CMA_3_ and DAPI were used in a double-staining process. Distamycin A solution was added to the cytological preparation. This protocol followed the one proposed by Guerra and Souza [19], with some modifications. The slides were prepared through enzymatic digestion with 2% cellulase and 20% pectinase for 1 h; this was followed by maceration in a drop of 45% acetic acid and then by freezing in liquid nitrogen to remove the cover slip. The slides containing the cytological preparations were aged for 3 days at room temperature, after which 0.25 mg^− 1^ of CMA_3_ was added for 1 h; this was followed by washing with distilled water and air-drying. Next, 0.1 mg^− 1^ distamycin A was added for 30 min, followed by another round of washing with distilled water and air-drying. Finally, DAPI was added for 30 min, followed by a last round of washing in distilled water and air-drying. The slides were assembled with 20 × 20 mm cover slips with 1:1 glycerol/McIlvaine medium (*v*/v), plus 2.5 mM MgCl_2_. After the application of the double staining, the slides were aged for another three days before analysis with epifluorescence microscopy.

### Fluorescent in situ hybridization

The application of FISH was performed following the protocol developed by Souza et al. [20], with some modifications, such as eliminating the pretreatment of the slides and adding digestion with pepsin (for better interference of the cytoplasm and cellular walls). The cytological preparations were digested with RNAse (100 μg/ml) and washed twice in 2xSSC (salt, sodium citrate) for 5 min. Next, 50 μl HCl (10 mM) was added and incubated for 5 min at room temperature. After removal of the cover slip, 50 μL of pepsin solution was added (0.75 μL of pepsin and 49.25 μL HCl, 10 mM), and the slide was kept in a humid chamber at 37 °C for 20 min. Next, the following steps were carried out: two washes with 2xSSC (5 min each); incubation in 4% paraformaldehyde for 10 min; two washes with 2xSSC (5 min each); and dehydration in an alcoholic series (70% ethanol and 95% ethanol; 5 min each). The slide was then air-dried for at least 30 min. The hybridization mixture was composed of 100% formamide (7.5 μL), 50% dextran (3.0 μL), 20xSSC (1.5 μL), 10% sodium dodecyl sulfate (0.2 μL) and the 45S probe (2.8 μL). This mix was heated in a thermocycler at 75 °C for 10 min, transferred to ice for at least 2 min and then placed on a slide, which was then denatured in a thermocycler at 75 °C for 10 min and placed in a humid chamber at 37 °C overnight. For the post-hybridization baths, the slide was washed with 2xSSC at room temperature, followed by two washes in 2xSSC at 42 °C (5 min each), two washes in 0.1xSSC at 42 °C (5 min each), two washes in 2xSSC at 42 °C (5 min each) and one wash in 4xSSC/0.2% Tween20 at room temperature (5 min). For detection, 50 μl of 5% BSA was applied to the slide for 10 min at room temperature, an antibody solution containing 0.7 μL of avidin-fluorescein isothiocyanate and 19.3 μL of 5% BSA was then added to the slide, which was kept in a dark, humid chamber for 1 h at 37 °C. Three washes were performed in 4xSSC/0.2% Tween 20 at room temperature, while still in the dark. Excess 4xSSC/0.2% Tween 20 was removed with a blade rinse in 2xSSC, and slide assembly was completed with 15 μL of DAPI-conjugated Vectashield® (Vector® Laboratories). The slides were refrigerated in a dark container for at least 24 h. Blade analysis was performed using the Olympus® BX41 fluorescence microscope; the images were captured with a DP25 digital camera and DP2-BSW software from Olympus®. The overlap of the images and the drawing of the boards were completed using Adobe Photoshop® software.

### Analysis of the 2C DNA

Five plants of each analyzed species were used in the analysis of the 2C DNA. For this, five leaves of each species were sampled for the analysis. The species *Zea mays* CV Kukurice (with 2C = 5.43 pg of DNA) and *Glycine max* L. (with 2C = 2.50 pg of DNA) [21] were used as internal standards to estimate the species’ genome sizes. The *Zea mays* species was used as an internal standard for the DNA content of all species except *Poincianella pluviosa*, for which *Glycine max* was used as the standard. Suspensions of intact nuclei were prepared using the Cystain PI Absolut P kit (Partec®). About 17 mg of leaf tissue from the target species and 20 mg of leaf tissue from the standard were minced simultaneously on a slide in Petri dish with 1 mL of extraction buffer. The suspension material was filtered through a nylon mesh screen of 50 μm. Then, 2 mL of solution containing RNAse and propidium iodide was added, and the material was incubated in a light-protected vessel for at least 30 min at room temperature. The evaluation of the 2C nuclear DNA was conducted using the Partec® PAII flow cytometer. The gain parameter was adjusted so that the peak for the nuclei of target species G1 was positioned over channel 50. At least 10,000 nuclei were analyzed for each sample. The fluorescence intensity of the nuclei, after staining with propidium iodide, was analyzed at rates of 20-50 nuclei/s. The positions of the peaks, their areas and their coefficients of variation were obtained from the cytometer. The size of the nuclear genome was calculated according to Dolezel [22]:

$$ 2\mathrm{C}\mathrm{DNA}=\frac{\mathrm{Average}\ \mathrm{peak}\ \mathrm{G}0/\mathrm{G}1\ \mathrm{for}\  Caesalpinia}{\mathrm{Average}\ \mathrm{peak}\ \mathrm{G}0/\mathrm{G}1\ \mathrm{for}\ \mathrm{standard}}\mathrm{x}\ 2\mathrm{C}\ \mathrm{DNA}\ \mathrm{standard}\ \left(\mathrm{pg}\right) $$

Analysis of variance (ANOVA) was used for the evaluation of significant differences in the flow cytometry data using a completely randomized design and five repetitions for each species. Additionally, the mean of the 2C DNA was clustered using Scott-Knott clustering. The ANOVA and the mean clustering were done using Sisvar software [23]. The editing of the Partec® flow cytometer histograms was carried out using Corel Draw® X7 software.

## Results

### Localization of GC-rich heterochromatin

**Fig. 1:**
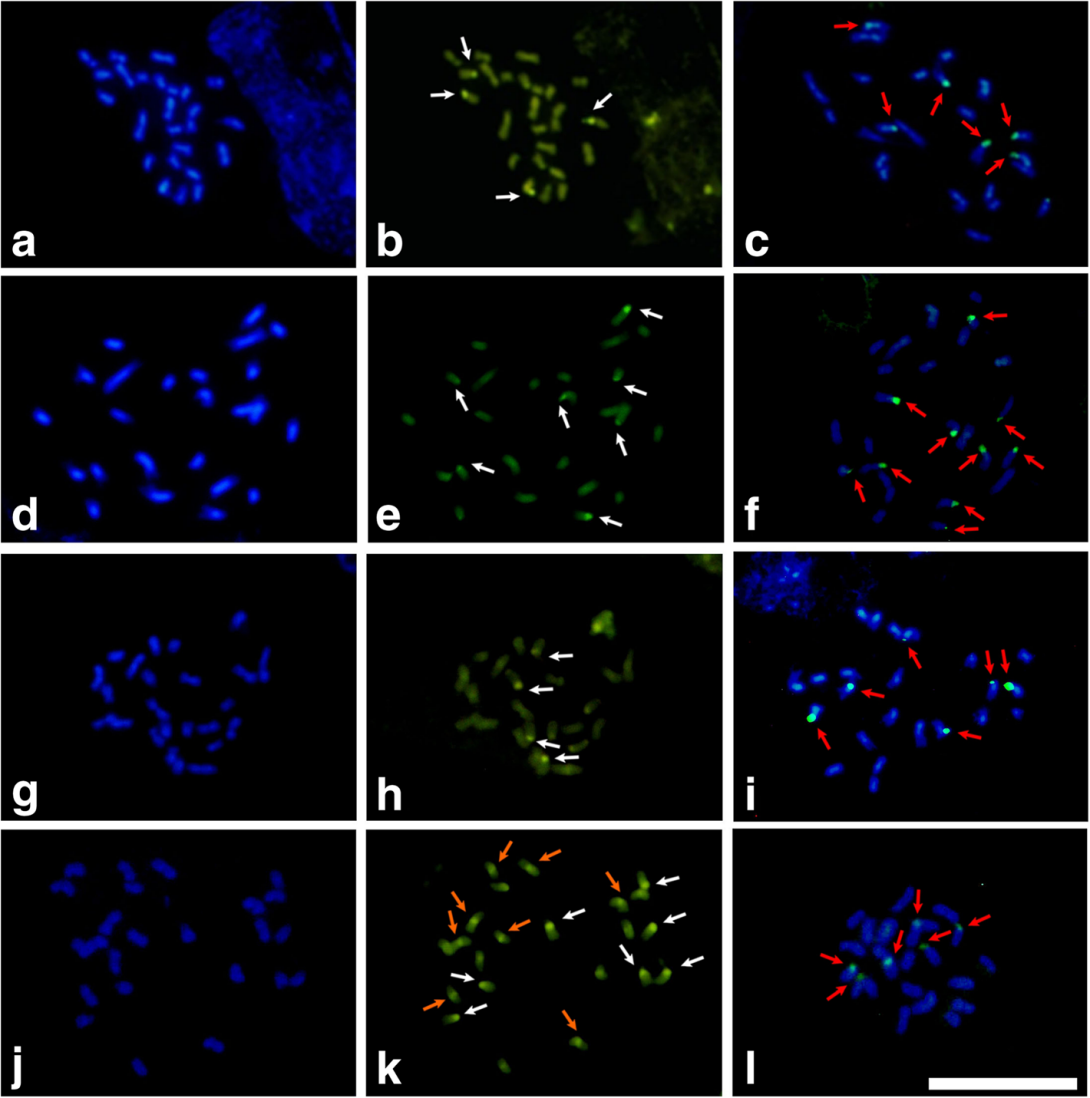
Application of fluorochromes in the *Caesalpinia* group. The fluorochromes DAPI (**a**, **d**, **g**, **j**) and CMA_3_ (**b**, **e**, **h**, **k**), as well as the FISH (**c**, **g**, **i**, **l**) with a probe for 45S rDNA on metaphase chromosomes. **a** - **c** *Cynostigma macrophyllum*, (**d** - **f**) *Erythrostemon calycina*, (**g** - **i**) *Poincianella pluviosa* and (**j** - **l**) *Libidibia ferrea*. White arrows indicate CMA_3_^+^/DAPI^−^ blocks, orange arrows indicate CMA_3_^+^ blocks, and red arrows indicate 45S rDNA sites; a bar is 10 μm

**Fig. 2:**
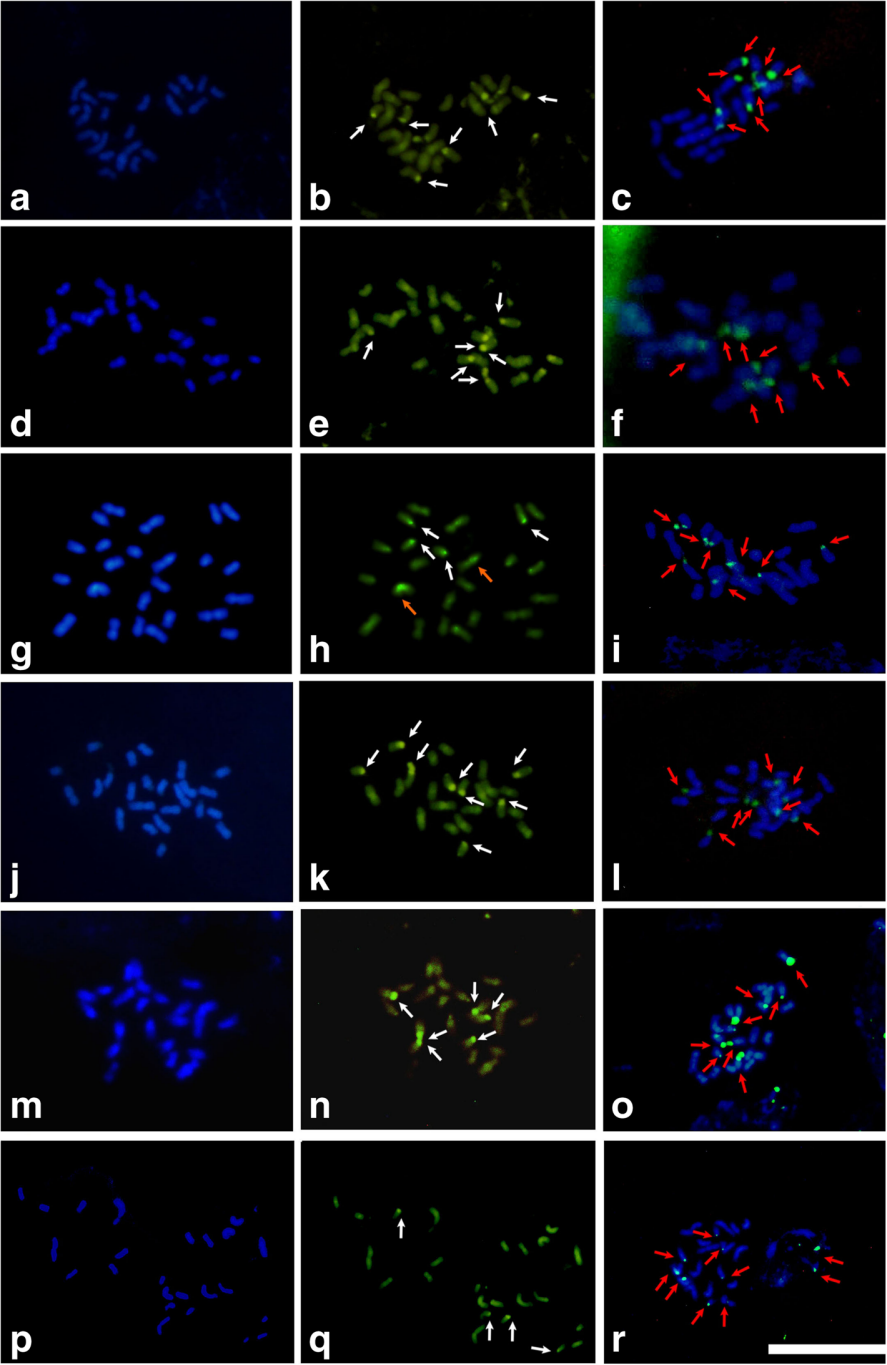
Application of fluorochromes in the *Caesalpinia* group. The fluorochromes DAPI (**a**, **d**, **g**, **j**, **m**, **p**) and CMA_3_ (**b**, **e**, **h**, **k**, **n**, **q**), as well as the FISH (**c**, **g**, **i**, **l**, **o**, **r**) with a probe for 45S rDNA on metaphase chromosomes. **a** - **c** *Poincianella bracteosa*, (**d** - **f**) *Po. laxiflora,* (**g** - **i**) *Po. microphylla*, (**j** - **l**) *Po. pyramidalis* (**m** - **o**), *Caesalpinia pulcherrima* and (**p** - **r**) *Paubrasilia echinata* (SV). White arrows indicate CMA_3_^+^/DAPI^−^ blocks, orange arrows indicate CMA_3_^+^ blocks, and red arrows indicate 45S rDNA sites

The location of GC-rich heterochromatin using base-specific fluorochromes revealed CMA_3_^+^/DAPI^−^ terminal blocks in all analyzed species. However*,*CMA_3_^+^ pericentromeric blocks were also observed in metaphase chromosomes in *Libidibia ferrea* and *Po. microphylla* (Figs. 1 and 2). In general, the terminal heterocyclic blocks were of distinct sizes. CMA_3_^+^/DAPI^−^ terminal blocks were observed in two chromosome pairs of *Cenostigma macrophyllum, Po. pluviosa, Caesalpinia pulcherrima* and *Pa. echinata*. Three chromosome pairs with CMA_3_^+^/DAPI^−^ terminal blocks were observed in *Po. bracteosa, Po.laxiflora, Po. microphylla* and *L. ferrea*. *Erythrostemon calycina* had seven chromosomes with CMA_3_^+^/DAPI^−^ terminal blocks. The highest number of CMA_3_^+^/DAPI^−^ terminal blocks was observed in *Po. pyramidalis*, which had four chromosome pairs with GC-rich heterochromatic blocks.

### Location of 45S rDNA sites

The application of FISH to localize the 45S rDNA sites allowed for the visualization of 45S rDNA in pairs of chromosomes (three, four or five, depending on the species), along with terminal 45S rDNA hybridization sites (Figs. 1 and 2). The karyotypes of the species *C. macrophyllum*, *Po. pluviosa* and *L. ferrea* each had three chromosome pairs with 45S rDNA hybridization sites (Fig. 1). The species *Po. bracteosa, Po. laxiflora, Po. microphylla,* Ca. *pulcherrima* and *Po. pyramidalis* each had four chromosome pairs with 45S rDNA sites (Fig. 2). Five chromosome pairs with hybridization sites for 45S rDNA were observed in *E. calycina* (Fig. 1f) and *Pa. echinata* (Fig. 2r).

All species demonstrated 45S rDNA hybridization sites in the fourth chromosome pair, and only Ca. *pulcherrima* did not show a marking on the seventh chromosome pair. The 45S rDNA hybridization sites in the eighth and tenth chromosome pairs were limited to *Po. microphylla* and *Po. pyramidalis*, respectively. The most frequent markers were located in the second chromosome pair (*E. calycina, L. ferrea* and *Po. pyramidalis*), in the fifth chromosome pair (*E. calycina, Po. bracteosa* and *Po. microphylla*) and in the eleventh chromosome pair (*E. calycina, Pa. echinata* and Ca. *pulcherrima*).

### Analysis of the 2C DNA

**Fig. 3:**
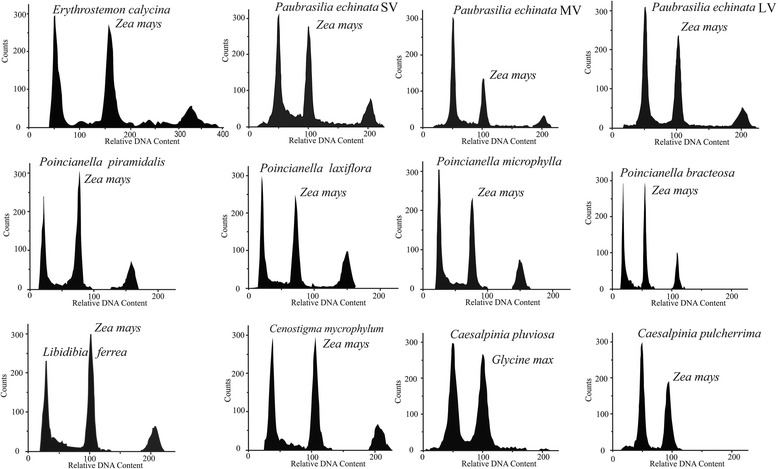
Histograms with 2C DNA content for species of the *Caesalpinia* group. Internal standards used: *Glycine max* (L.) Merr and *Zea mays* L

The histograms in Fig. 3 show the fluorescence distribution as a function of the number of nuclei in the examined sample (Fig. 3). The *Pa. echinata* large-leaf variant (LV) was the taxon with the highest 2C value of DNA (2.82 pg). The *Pa. echinata* LV was then used as the reference for high DNA content; compared to this reference value, the DNA content of the other species was lower by 46.3% for *L. ferrea*, 45.4% for *E. calycina*, 42.2% for Ca. *pulcherrima*, 35.1% for *C. macrophyllum*, 33.7% for *Po. microphylla*, 33.3% for *Po. pluviosa*, 32.6% % for *Po. laxiflora*, 31.9% for *Po. bracteosa* and *Po. pyramidalis*, 2.1% for the *Pa. echinata* small-leaf variant (SV), and 0.4% for the *Pa. echinata* medium-leaf variant (MV).

The ANOVA for the 2C DNA of the species revealed a highly significant difference, with a low coefficient of variation: 1.45% (Table 2). The Scott-Knott test clustered the taxa into six groups based on the average 2C values, with a minimum significant difference of 0.0647 (Table 1). Species from the genus *Poincianella* were arranged into group C. Two species remained in isolated groups: *E. calycina* and *C. macrophyllum*. The species *L. ferrea* and Ca. *pulcherrima* were placed in group E.

**Table 2:** Summary of the ANOVA for the characteristic 2C DNA content among the analyzed species

Sources of variation	GL	QM
Taxon	11	1.136674^a^
Error	48	0.000888
CV (%)	1.46	

^a^significant at 1% probability by F test

*GL* Level of freedom, *QM* Average square, *CV* Coefficient of variation

An ANOVA was carried out to estimate the variation of the DNA content for only the three morphotypes of *Pa. echinata*, thus demonstrating the existence of a significant difference at *p-value* = < 0.05 (Table 3). The Scott-Knott test with only the morphotypes of *Pa. echinata* showed a separation between the morphotypes, as the SV type was isolated in group B with a statistically significant difference in relation to the MV and LV types in group A.

**Table 3:** Summary of the ANOVA for 2C DNA content among the three morphological leaf variants of *Paubrasilia echinata*

Sources of variation	GL	QM
Taxon	2	0.012653^a^
Error	12	0.012640
CV (%)	1.16	

^a^significant at 5% probability by F test

*GL* Level of freedom, *QM* Average square, *CV* Coefficient of variation

## Discussion

Prior studies have been carried out to locate heterochromatin rich in AT and GC among species from the *Caesalpinia* group using the fluorochromes CMA_3_ and DAPI. Previous studies in the *Senna* and *Chamaecrista* genera revealed the presence of CMA_3_^+^/DAPI^−^ and small CMA_3_^−^/DAPI^+^ terminal or subterminal blocks [14]. In *Copaifera*, only CMA_3_^+^/DAPI^−^ blocks were observed [24], similar to the terminal pattern observed in the present study, for which the analyzed species showed heterochromatin blocks that were rich in GC and poor in AT. These CMA_3_^+^/DAPI^−^ bands have been observed only in the terminal regions of chromosomes and have ranged from two to four pairs of chromosomes in the analyzed species. Additionally, CMA_3_^+^ blocks have been observed in the metaphase chromosomes of *L. ferrea* and *Po. microphylla*, indicating either the existence of GC-rich pericentromeric sequences in related genera (suggesting a shared trait with a common ancestor) or changes related to the composition of centromeric satellite DNA, which is qualitatively rich in GC in these species.

Fluorochrome CMA_3_^−^/DAPI^+^ blocks have already been observed in *Senna obtusifolia* (L.) H.S. Irwin & Barneby and in one population of *Chamaecrista nictitans* Moench [14]. However, these bands were not reported in the present study. The absence of this type of heterochromatin may be a typical characteristic of species in the analyzed genera of *Poincianella, Libidia, Erytrostemon, Paubrasilia, Caesalpinia* and *Cenostigma*. However, the karyotype characterization on more population can relieve the interspecific variation in cytogenetic markers. In the Leguminosae family, various patterns of AT-rich and GC-poor heterochromatin were also observed for the *Mimosa* [25] and *Erythrina* [26] genera. These variations reinforce the need to characterize heterochromatin in other species so as to understand the distribution pattern and evolution of this class of DNA, which is variously colored within the *Caesalpinia* group.

In the present study, CMA_3_^+^/DAPI^−^ terminal blocks coincided with certain 45S rDNA hybridization sites, reinforcing the fact that these CMA_3_^+^ blocks relate to these 45S rDNA, which in turn are rich in GC bases [27–29]. However, the application of base-specific fluorochromes did not reveal rDNA sites with few repetitions, as the small heterochromatic block made detection and photographic documentation unviable for the epifluorescence microscope [30].

Molecular cytogenetics techniques have been widely used to localize specific in situ DNA sequences [31]. Genetically related species tend to have karyotypes with similar characteristics in terms of sequence localization and are useful in studies of plant systematics, taxonomy and evolution mainly contributing to groupings of species or cytotypes that share common characteristics, thus suggesting primitiveness or deactivation for a given cytological marker that is shared among a group of plants [32–38].

The hybridization sites of 45S rDNA probes in species of the *Caesalpinia* group have shown variations in both the number and the location of these sequences. The differences in the number of such sequences generally occur due to chromosomal rearrangements such as translocations, inversions, duplications and deletions [39], whereas variations in the signal intensity of hybridization are observed between sites with different numbers of rDNA replicates. Any changes in these sites’ patterns of distribution are levels of speciation; this may assist in determining how evolution has occurred within a group of taxonomically complex plants [31, 40]. Lower quantitative variation has been observed in the *Poincianella* genus, suggesting greater stability in the number of 45S rDNA sites (a total of eight). Conservation in the location of the rDNA genes (as revealed using FISH) was observed for species from the genus *Trifolium* (Leguminosae: Papilionoideae), which may indicate that some Leguminosae have great stability in this region [41]. This stability, which is based on the number and location of a chromosomal marker, is a good characteristic for species identification and delimitation through karyotype analysis.

The species in this study showed significant variations in 2C DNA and, consequently, in the size of the genomes for the evaluated species and genera. In addition, the low coefficient of variation among the replicates indicates the precision of the sample, as analyzed using flow cytometry. The variation in the amount of DNA across species can be attributed to the loss or gain of DNA sequences, which usually consist of repetitive DNA; this may occur due to evolutionary changes in accumulation and/or loss of repeating monomers in the micro and macro environments during the species’ evolution [42, 43]. This suggests that such losses or additions to the genome become stabilized during microevolution and selection [43].

In this work, only Ca. *pulcherrima* had a lower estimated amount of 2C DNA (1.63 pg) than its previous estimate (1.80 pg) [16]. This may be due to the variation in number of chromosomes for the two analyzed populations of Ca. *pulcherrima*, as the population evaluated in this study presented 2*n* = 24, but the population evaluated by OHRI et al. [16] presented 2*n* = 28. The estimated DNA nuclear content for diploid Ca. *crista* (2*n* = 24) indicated a too-high value of 0.707 pg per chromosome, which leads to 17.67 pg for the 2C DNA. This value, which is much higher than our result, can be attributed to the different cytophotometric methods, as *Allium cepa* L.’s DNA value was computed as a DNA size pattern [17]. The 2C value that we found for Ca. *crista* was also considerably higher than those that OHRI et al. [16] found for the species in the genus Caesalpinioideae. Analyzing the DNA content and chromosomal differences observed for taxa from the *Caesalpinia* genus, together with the results from the literature, requires an interdisciplinary mode in order to indicate the species’ taxonomy, delimitation and clustering.

Many species in the *Caesalpinia* group exhibit a high degree of phenotypic plasticity, especially in foliage and leaflets. This has resulted in multiple nomenclature for the species, with each leaf-size variant having a specific condition, thus resulting in taxonomic problems [1, 5, 7, 9]. This fact can be observed in *Pa. echinata*, which was previously arranged in the *Caesalpinia* genus and which has three morphotypes that were previously characterized using chloroplast DNA sequences [44]. The three morphotypes (leaf-size variants) presented small variations in 2C DNA, with values of 2.76, 2.81 and 2.82 pg for the SV, MV and LV types, respectively. The Scott-Knott test separated these morphotypes into two groups, one with only *Pa. echinata* SV and one composed of *Pa. echinata* MV and LV; this shows that, although the variations among *Pa. echinata* leaf-size morphotypes are not large, the values are sufficient to separate the SV morphotype from the other two variants, with this type’s low DNA content acting as a differentiating feature.

In legume species, a positive correlation has been observed between leaf size and nuclear DNA content [45]. This relationship was also observed in this study, wherein the variants with the relatively large leaves (MV and LV) had more DNA than the SV variants. Therefore, diversification of genome size results from speciation, which, along with phenotypic changes in quantitative descriptors, is an adaptation response such as the ones observed in polyploid plants [46]. Thus, plants’ 2C DNA can be used to estimate the taxonomic differentiation between species, as seen here for the variants of *Pa. echinata*.

The data obtained in our studies corroborate the new classification of the species that were initially placed in the *Caesalpinia* group [5, 9–11], showing that the species that had at least 1.87 pg of 2C DNA in this study should actually be grouped in the *Poincianella* genus. Among the species analyzed in the present study, the only representative of the *Erythrostemon* genus was *E. calycina*, which had the least amount of 2C DNA (1.54 pg) and which was also the only species to present five pairs of chromosomes with the presence of 45S rDNA sites; these were unique characteristics of this genus. In previous analyses, similarities in chromosome morphology have been reported for the six species studied herein, and karyotype formulas have shown the predominance of metacentric chromosomes [47].

Only one species was evaluated for the *Cenostigma* genus, *C. macrophyllum*; this species which was initially collected under the belief that it belonged to the *Caesalpinia* genus. The similarities between species of these two genera have also been visualized previously, as the species *Cenostigma sclerophyllum* Tul. was later described as a synonym for *Caesalpinia marginata* Tul. [8, 48]. The analyses of *C. macrophyllum* enabled us to observe that it was the only species to present only six CMA_3_^+/^DAPI^−^ bands, showing that the amount of GC-rich heterochromatin was lower than that of the other species evaluated herein; this could be a feature exclusive to the *Cenostigma* genus.

In this work, the distribution pattern for heterochromatin, the physical location of 45S rDNA regions and the amount of DNA were all useful to corroborate studies of systematics and of evolution in *Caesalpinia*-group species. Although the quantity of species evaluated herein is only a small fraction of the diversity already described as belonging to the *Caesalpinia* group, and although some species were relocated within new genera, it was possible to observe a distinctive pattern for individual cytogenetic characteristics in the genera currently specified as *Poncianella*. This shows that the karyotypic analysis and the quantification of 2C DNA are valid methods to support taxonomic and biosystematics studies.

## Conclusion

The quantitative variation in GC-rich heterochromatin among species in the *Caesalpinia* group indicates not only the variable number of satellite-related rDNA sites but also the existence of chromosomes with pericentromeric repetitive DNA with GC-rich heterochromatin in *L. ferrea* and *Po. microphylla* species. The intra- and interspecific variations in size of the GC-rich chromosomal blocks related to rDNA and satellites (relative to the location of these regions), as determined using the FISH technique with a probe for 45S rDNA. This relation suggests that the use of fluorochromes for the localization of 45S rDNA is not indicated for *loci* numbers identification in species of the *Caesalpinia* group. This fact is attributed to the minor size replications of the 45S rDNA genes in some chromosomes, which make it difficult to observe variations using CMA_3_ fluorochromes. The 2C DNA may not be even related to morphological leaf size for *Paubrasilia echinata*, so this relationship must be evaluated again with other populations to increase the number of analyzed plants. On the other hand, the 2C DNA helped us to see all the variation in the *Caesalpinia* group within this trait. All the data indicate that the actual taxonomy appropriated by the *Caesalpinia* group is due to the larger chromosomal and genome-size variations, which could be clustered in a specific genus. This information shows the group from point of view that differs from the old taxonomy, which grouped all species analyzed herein as just one genus.

## Abbreviations

2C DNA: 2C DNA content
2n: Diploid number
ANOVA: Analysis of variance
CMA_3_: Chromomycin A_3_
DAPI: 4′-6-diamidino-2-phenylindole
FISH: Fluorescent in situ hybridization
LV: Large-leaf variant
MV: Medium-leaf variant
rDNA: Ribosomal DNA
SSC: Salt sodium citrate
SV: Small-leaf variant
